# Regular Practice of Physical Activity Improves Cholesterol Transfers to High-Density Lipoprotein (HDL) and Other HDL Metabolic Parameters in Older Adults

**DOI:** 10.3390/nu15234871

**Published:** 2023-11-22

**Authors:** Pedro G. S. Braga, Fatima R. Freitas, André L. L. Bachi, Gislene R. Amirato, Roberta V. Baroni, Maria Janieire N. N. Alves, Rodolfo P. Vieira, Mauro W. Vaisberg, Marlene N. Aldin, Roberto Kalil Filho, Antônio M. Figueiredo Neto, Nágila R. T. Damasceno, Thauany M. Tavoni, Raul C. Maranhão

**Affiliations:** 1 Instituto do Coracao, Hospital das Clinicas HCFMUSP, Faculdade de Medicina, Universidade de Sao Paulo, Sao Paulo 05403-900, Brazil; pedro.senger@gmail.com (P.G.S.B.); robertavbaroni@gmail.com (R.V.B.); thauany.martins@alumni.usp.br (T.M.T.); 2 Post-Graduation Program in Health Sciences, Santo Amaro University (UNISA), Sao Paulo 04829-300, Brazil; 3 Centro Educacional e Esportivo Mane Garrincha, Secretaria Municipal de Esportes, Lazer e Recreacao, Sao Paulo 04039-034, Brazil; 4 Post-Graduate Program in Sciences of Human Movement and Rehabilitation, Universidade Federal de Sao Paulo (UNIFESP), Santos 11060-001, Brazil; 5 Post-Graduate Program in Human Movement and Rehabilitation and in Pharmaceutical Sciences, Evangelical University of Goiás (Unievangélica), Anápolis 75083-515, Brazil; 6 Post-Graduate Program in Bioengineering, Universidade Brasil, São Paulo 08230-030, Brazil; 7 Departamento de Medicina, Universidade Federal de Sao Paulo (UNIFESP), Sao Paulo 04023-062, Brazil; vaisberg.mauro@gmail.com; 8 Departamento de Nutricao, Faculdade de Saude Publica da Universidade de São Paulo (FSP-USP), Sao Paulo 01246-904, Brazil; 9 Instituto de Fisica (IF), Universidade de Sao Paulo, Sao Paulo 05508-090, Brazil; afigueiredo@if.usp.br; 10 Faculdade de Ciencias Farmaceuticas, Universidade de Sao Paulo, Sao Paulo 05508-000, Brazil

**Keywords:** aging, cholesterol, HDL metabolism, HDL function, physical activity

## Abstract

The effects of regular physical activity on two important anti-atherosclerosis functions of high-density lipoprotein (HDL), namely its capacity to receive both forms of cholesterol and its anti-oxidant function, were investigated in this study comparing older adults with young individuals. One-hundred and eight healthy adult individuals were enrolled and separated into the following groups: active older (60–80 yrs, n = 24); inactive older (60–79 yrs, n = 21); active young (20–34 yrs, n = 39); and inactive young (20–35 yrs, n = 24). All performed cardiopulmonary tests. Blood samples were collected in order to assess the following measures: lipid profile, HDL anti-oxidant capacity, paraoxonase-1 activity, HDL subfractions, and lipid transfer to HDL. Comparing active older and active young groups with inactive older and inactive young groups, respectively, the active groups presented higher HDL-C levels (*p* < 0.01 for both comparisons), unesterified cholesterol transfer (*p* < 0.01, *p* < 0.05), and intermediate and larger HDL subfractions (*p* < 0.001, *p* < 0.01) than the respective inactive groups. In addition, the active young group showed higher esterified cholesterol transfer than the inactive young group (*p* < 0.05). As expected, the two active groups had higher VO_2_peak than the inactive groups; VO_2_peak was higher in the two younger than in the two older groups (*p* < 0.05). No differences in unesterified and esterified cholesterol transfers and HDL subfractions were found between active young and active older groups. HDL anti-oxidant capacity and paraoxonase-1 activity were equal in all four study groups. Our data highlight and strengthen the benefits of regular practice of physical activity on an important HDL function, the capacity of HDL to receive cholesterol, despite the age-dependent decrease in VO_2_peak.

## 1. Introduction

The transfer of cholesterol to high-density lipoprotein (HDL) in either unesterified or esterified forms from the other lipoprotein classes, such as very-low density lipoprotein (VLDL) or low-density lipoprotein (LDL), continuously occurs in the plasma compartment [1]. Unesterified cholesterol transferred to HDL is esterified by the action of lecithin–cholesterol acyltransferase (LCAT) using apolipoprotein A-I, the main HDL protein, as a co-factor. Unesterified cholesterol is thereby sequestered from HDL particles’ surfaces to HDL cores [2]. Esterified cholesterol in the apolipoprotein B-containing lipoproteins can be transferred to HDL by the action of cholesteryl ester transfer protein (CETP) and stored in the lipoprotein core. The esterified cholesterol pool in HDL is eventually taken up by the liver and excreted in the bile. Alternatively, HDL esterified cholesterol can be transferred to the apolipoprotein B-containing lipoproteins by the action of CETP. Those fluxes are essential for the homeostasis of cholesterol in the plasma and are also important for reverse cholesterol transport. In reverse cholesterol transport, unesterified cholesterol pumped out from the cells of peripheral tissues is transferred to HDL, where it is esterified and undergoes the above-described cholesterol fluxes among lipoproteins [1].

Previously, we showed through an in vitro assay that reduced cholesterol transfer from a model lipoprotein to HDL is related with the presence of atherosclerotic diseases or conditions that facilitate atherosclerosis development [3,4]. In this respect, lower cholesterol transfer to HDL occurred in physically inactive individuals, whereas physical activity increased those transfer rates. The effects of physical activity on cholesterol transfer to HDL were elicited regardless of the modality of training [5,6].

Physical activity is a major non-pharmacologic resource to achieve improvement in lipid profile, especially through increased HDL cholesterol (HDL-C) levels, including in the older adult population [6,7]. In this regard, we previously showed that older women who regularly engaged in exercise training not only presented higher HDL-C levels but also increased cholesterol transfer to HDL as compared to a group of sedentary older women [6]. Nonetheless, higher HDL-C is not necessarily accompanied by higher cholesterol transfer rates to HDL. For example, in patients with metabolic syndrome, exercise training elicited an increase in cholesterol transfer without increasing HDL-C [5].

In a previous study, we observed that older women engaging in regular exercise had a higher capacity to transfer cholesterol to HDL than sedentary women [6]. That finding encouraged us to test the process of cholesterol transfer in both genders and in different age ranges in an attempt to acquire a novel global parameter to assess the metabolic effects of regular exercise. A second test of HDL’s functional properties, specifically involving its antioxidant action on LDL lipids, was also performed to investigate whether more than one HDL property could be simultaneously changed by the stimulus of exercise practice. Other parameters related to HDL metabolism, such as CETP and LCAT concentrations, the activity of paraoxonase 1 (PON-1), HDL size and subfractions, and lipid and apolipoprotein plasma profiles, were determined, aiming to establish correlations with the functional properties of HDL.

## 2. Materials and Methods

### 2.1. Participants

This study evaluated older (aged > 60 yrs) and young adults (20–35 yrs) of both sexes selected from the Mané Garrincha Sports Education Center of the Department of Sports of São Paulo City, Brazil, and from the employees and students of the Heart Institute of the University of São Paulo Medical School Hospital (InCor-HCFMUSP). All the enrolled participants were thoroughly inquired about their physical activity routine, specifically whether they were engaged or not in an exercise training program for at least the last twelve months [8]. Based on their answers, they were allocated to four study groups: active older (n = 24), inactive older (n = 21), active young (n = 39), and inactive young (n = 24) individuals. The selected volunteers were then submitted to a treadmill exercise test. The predicted VO_2_peak for age was used to confirm the inclusion by the inquiry of each participant in the active or in the inactive group.

Dietary habits were not scrutinized in the inclusion inquiry, and no recommendations on food consumption before blood sampling were made in addition to the required 12 h fasting. None of the participants were smokers, addicted to alcohol consumption, or were using lipid-lowering drugs, beta-blockers, or anabolic steroids. The exclusion criteria were as follows: individuals with diabetes, previous cardiac or cerebrovascular events, pulmonary, cardiovascular, renal, metabolic, inflammatory, or neoplastic diseases.

The study was conducted in accordance with the Declaration of Helsinki and Ethical Standards in Sport and Exercise Science Research [9] and approved by the Institutional Review Board and Ethics Committee of the Hospital das Clínicas da Universidade de São Paulo (protocol code no. 2.903.252; date of approval 19 September 2018). A written informed consent was obtained from all individuals after a complete description of the protocol.

### 2.2. Cardiopulmonary Exercise Test

The test was performed on a programmable treadmill (T2100 Model, GE Healthcare, Chicago, IL, USA) using a ramp protocol with increments in workload each minute until volitional exhaustion [9]. Oxygen uptake was measured in a cardiopulmonary exercise test breath-to-breath to determine cardiorespiratory fitness by the VO_2_peak (SensorMedics—Vmax Analyzer Assembly, Palm Springs, CA, USA). VO_2_ ventilatory threshold, absolute VO_2_, and VO_2_peak were determined [10]. Age-predicted VO_2_ was calculated according to gender [11], metabolic equivalent (MET) of peak [12], and oxygen uptake efficiency slope (OUES) [13]. The effectiveness of the cardiopulmonary test was assessed and included those reaching values of respiratory exchange ratio (VO_2_/VCO_2_) equal to or higher than 1 [9] and by the request of the participant to stop the test by gestures, which indicate maximal effort. All subjects were motivated for maximal performance in the test, and none showed signals of myocardial ischemia during the exercise test.

### 2.3. Blood Sampling, Plasma Lipids, and Apolipoproteins

Blood samples were collected after fasting 12 h overnight in tubes containing EDTA anticoagulant (ethylenediamine tetra-acetic acid) or not, and plasma/serum samples were obtained after centrifugation (300× *g*, 10 min at 4 °C).

Plasma lipids were determined by the colorimetric–enzymatic method (Merck KGaA, Darmstadt, Germany). LDL-C was estimated by the Friedewald formula [14]. Apolipoproteins were measured by the immunonephelometric method (ProSpec-Tany TechnoGene Ltd., Rehovot, Israel).

### 2.4. HDL Size and Subfractions

The particle size of HDL was measured in fresh plasma after chemical precipitation of apolipoprotein B-containing lipoproteins by the laser light scattering method (Malvern Instr., Worcestershire, UK) [4]. The cholesterol content of the HDL subfractions was analyzed by electrophoresis (Lipoprint System Quantimetrix Corporation, Redondo Beach, CA, USA).

### 2.5. CETP and LCAT Concentrations

The plasma concentrations of CETP and LCAT were determined by ELISA immunoassays (ALPCO Diagnostics, Salem, MA, USA).

### 2.6. Paraoxonase 1 (PON1) Activity

PON1 activity was measured by adding 1M Tris-HCl buffer (100 mmol/L, pH 8.0) containing 2 mmol/L CaCl_2_ and 5.5 mmol/L paraoxon (Sigma Chemical Company, St. Louis, MO, USA) to the serum samples. The generation of p-nitrophenol was measured at 405 nm at 37 °C in a plate reader (Victor X3, PerkinElmer, Shelton, CT, USA) [15].

### 2.7. HDL’s Antioxidant Capacity

HDL’s antioxidant capacity was determined by a modified lag time method using CuSO_4_ as the oxidizing agent [16]. This method uses standard LDL purified by ultracentrifugation. HDL was precipitated using phosphotungstic acid and magnesium chloride (HDL Cholesterol kit, Labtest, Minas Gerais, Brazil). LDL (83 ug protein/mL), HDL (200 ug protein/mL), and CuSO_4_ (30 μM) were incubated. The antioxidant capacity of HDL was monitored at 234 nm for 5 h at 37 °C. The following parameters were calculated: lag time; DOmax, defined as the maximum production of conjugated dienes; Tmax, the time needed to achieve DOmax; Vmax, defined as the maximum rate of conjugated dienes; and time to Vmax, the time needed to achieve Vmax.

### 2.8. Cholesterol Transfer Assay

A nanoemulsion was prepared from a lipid mixture composed of 40 mg cholesteryl oleate, 20 mg egg phosphatidylcholine, 1 mg triolein, and 0.5 mg cholesterol (Sigma Chemical Co.), as described previously [3,4]. Trace amounts of 4-14C-cholesterol or [1α,2α(n)-3H]-cholesteryl oleate (Amersham, Little Chalfont, Buckinghamshire, UK) were added to the initial solution. The emulsification of lipids by prolonged ultrasonic irradiation in aqueous media and the two-step procedure of ultracentrifugating the crude emulsion and adjusting its density with KBr to obtain a nanoemulsion were carried out.

The in vitro assay to measure cholesterol transfer from the nanoemulsion to HDL has been previously described [3,4]. Briefly, the nanoemulsion is incubated with plasma. This incubation is followed by chemical precipitation of the nanoemulsion and apolipoprotein B-containing lipoproteins. The radioactivity of the supernatant containing HDL is measured by a liquid scintillation counter (Liquid Scintillation Analyzer Tri-Carb2100TR, PerkinElmer). The transfer of cholesterol to HDL is expressed as a percentage of the total radioactivity of the nanoemulsion incubated with the plasma.

### 2.9. Statistical Analysis

The normality and the homogeneity of variance of the data obtained in this study were determined by the Kolmogorov–Smirnov and Levene’s tests, respectively. Parametric data are expressed as mean ± standard deviation, and non-parametric data are expressed as median (minimum:maximum).

Categorical variables were analyzed by the chi-squared test, whereas numerical data were analyzed by using one-way ANOVA followed by multiple comparisons of Bonferroni or by using a Kruskal–Wallis test followed by multiple comparisons of Dunn.

Pearson or Spearman’s rank correlation coefficient tests were used to determine the occurrence of correlation among the variables assessed in this study. In addition, a multivariate regression analysis adjusted for BMI was performed.

Significance was set as *p* ˂ 0.05 for all analyses, and GraphPad Prism version 5.00 was used for statistical analysis.

## 3. Results

As shown in Table 1, body mass index (BMI) and waist circumference were higher in the inactive older group compared to the inactive young group but was equal to the active older group. BMI was also higher in the inactive older group than the active older group. Waist–hip ratio (WHR) was higher in the active older group than in the active young group and higher in the inactive older group than in the inactive young group.

VO_2_ ventilatory threshold, peak, age-predicted VO_2_, and MET were higher in the active older group than in the inactive older group. VO_2_ absolute values, ventilatory threshold, peak, age-predicted VO_2_, MET, and OUES were higher in the active young group than the inactive young group. VO_2_ absolute values, ventilatory threshold, peak, MET, and OUES were higher in the active young group in comparison to the active older group. VO_2_ of ventilatory threshold, peak, age-predicted VO_2_, and MET were higher in the inactive young group than the inactive older group.

As shown in Table 2, HDL-C was higher in the active older group than in the inactive older group and in the active young group than the inactive young group. LDL-C and non-HDL-C were higher in the active older group than in the active young group. Non-HDL-C, LDL-C, and triglycerides were higher in the inactive older group than in the inactive young group. Apolipoprotein A-I was similar between the active older group and the inactive older group but higher in the active young group than in the inactive young group. Apolipoprotein A-I was higher in the inactive older group than in the inactive young group. Apolipoprotein B concentration did not differ between the active older and inactive older groups or between the active young and inactive young groups. However, apolipoprotein B was higher in the active older group than the active young group and in the inactive older group than the inactive young group.

In Table 2, it is also shown that the transfer of unesterified cholesterol to HDL was higher in the active older group than in the inactive older group, but that of esterified cholesterol was equal. Transfers of unesterified and esterified cholesterol were higher in the active young group than in the inactive young group. CETP concentrations were higher in the active older group than the active young group. LCAT concentration and HDL particle size were not different between the groups (Table 2).

HDL anti-oxidant capacity and PON1 activity were equal among the study groups (Table 3).

**Figure 1:**
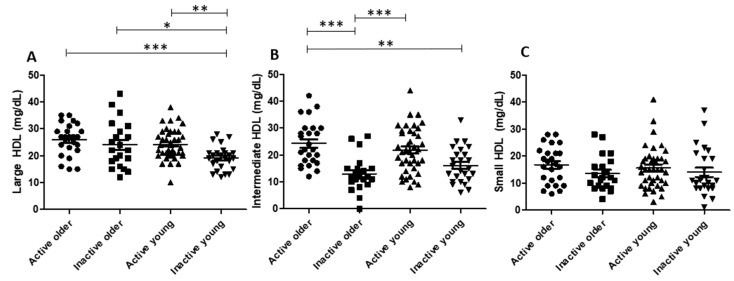
HDL subfractions in study groups. Large (**A**), intermediate (**B**), and small (**C**) HDL subfractions in study groups. Abbreviations: HDL: high-density lipoprotein. *: *p* < 0.05; **: *p* < 0.01; ***: *p* < 0.001.

Figure 1 shows the data of HDL subfractions. The larger HDL subfraction was equal in the active older group and the inactive older group. However, intermediate HDL was higher in the active older group than in the inactive older group. Moreover, larger HDL was higher in the active young group than in the inactive young group, without differences among groups regarding intermediate HDL. Additionally, there were no differences in HDL subfractions between the active older group and the active young group. Larger HDL was higher in the inactive older group than in the inactive young group. Groups had no differences in small HDL.

Supplementary Table S1 shows the correlation analysis performed with different parameters measured in the study. VO_2_peak was negatively correlated with concentrations of LDL-C (r = −0.3447; *p* < 0.001), non-HDL-C (r = −0.4109; *p* < 0.001), triglycerides (r = −0.3558; *p* < 0.01), apolipoprotein B (r = −0.3630; *p* = 0.001), and CETP (r = −0.2417; *p* < 0.05). Esterified cholesterol transfer was positively correlated with HDL-C (r = 0.4597; *p* < 0.001), LDL-C (r = 0.3024; *p* < 0.01), non-HDL-C (r = 0.3363; *p* < 0.001), triglycerides (r = 0.2597; *p* < 0.01), apolipoprotein A-I (r = 0.6168; *p* < 0.001), apolipoprotein B (r = 0.3159; *p* < 0.001), and unesterified cholesterol transfer (r = 0.8685; *p* < 0.001). Esterified cholesterol transfer was also positively correlated with the concentrations of CETP (r = 0.3137; *p* < 0.001) and LCAT (r = 0.3641; *p* < 0.001), HDL particle size (r = 0.3471; *p* < 0.001), and the intermediate (r = 0.3910; *p* < 0.001), and large HDL subfractions (r = 0.4447; *p* < 0.001). Unesterified cholesterol transfer to HDL was positively correlated with HDL-C (r = 0.5415; *p* < 0.001), apolipoprotein A-I (r = 0.6652; *p* < 0.001), HDL particle size (r = 0.3315; *p* < 0.001), and the concentrations of CETP (r = 0.2467; *p* < 0.01) and LCAT (r = 0.4060; *p* < 0.001). Unesterified cholesterol transfer also positively correlated with the small (r = 0.2365; *p* < 0.05), intermediate (r = 0.4843; *p* < 0.001), and large HDL subfractions (r = 0.4098; *p* < 0.001).

Since BMI could impact either metabolic or physical capacity parameters, we additionally performed a multivariate regression analysis adjusted for BMI. In this respect, the BMI observed in the active older group showed a significant effect on MET (β = −1.223; 95% CI −0.2278 to −0.1676; *p* = 0.0237; R2 = 0.9967); on larger HDL (mg/dL; β = 4.318; 95% CI 0.7100 to 7.926; *p* = 0.0263; R2 = 0.9978); and on intermediate HDL (mg/d; β = 4.207; 95% CI 0.9046 to 7.510; *p* = 0.0176; R2 = 0.9986). Moreover, the BMI observed in the young active group and in the young inactive group showed a significant effect on age-predicted VO_2_ (%; β = −0.2739; 95% CI −0.3856 to −0.1622; *p* < 0.0001; R2 = 0.8589; and β = −0.3581; 95% CI −0.6077 to −0.1084; *p* = 0.0096; R2 = 0.9459, respectively).

## 4. Discussion

Our data showed that several parameters of HDL metabolism assessed in the present study were better in active compared to inactive individuals, regardless of whether they were older (>60 yrs) or younger (20–35 yrs).

In this regard, an increase in HDL-C by regular participation in exercise, a classical all-age observation [17,18,19], was confirmed here, along with higher levels of intermediate or larger HDL subfractions in the two active groups compared to the two inactive groups. According to the literature, larger HDL subfractions are more protective against atherosclerosis than smaller subfractions [20,21], and exercise training has been associated with increments in larger HDL particles [22]. This finding was also reported in subjects with different morbidities that performed exercise training, such as men and women with overweight and obesity [22,23], as well as in young obese women: exercise training was able to reduce the small HDL subfraction [23]. Taken together, our finding of a higher intermediate HDL subfraction in the active older group than in the inactive older group and of increased larger HDL subfractions in the active young group compared to the inactive young group suggests that physical activity improves the quality of the HDL subfraction profile, either in young or older individuals.

Of note was the fact that in our study, the inactive older group had a higher large HDL subfraction than the inactive young group. This is somehow in disagreement with the description by Otrane et al. [24] that found higher large HDL and lower small HDL subfractions in younger compared to older subjects. These discrepant findings can putatively be related to dietary habits, since Otrane et al. [24] mentioned that adherence to a Mediterranean-type diet, and specifically the intake of extra virgin olive oil, was able to increase the large and intermediate HDL subfractions in older individuals. Here, we did not explore the dietary habits of the participants, which can be considered a limitation of this study.

The multivariate analysis yielded an unexpected correlation, i.e., the higher BMI and the higher presence of the large and intermediate HDL particle subfractions in the active older group. The large subfraction is considered the most protective subfraction, and higher BMI is consistently harmful to health.

Here, PON1 activity and HDL antioxidant capacity were not different among all groups. Previously, we had shown that patients with metabolic syndrome have increased PON1 activity after an exercise training program performed over 12 weeks [5]. Our finding that HDL antioxidant capacity was not higher in exercise practitioners is supported by a previous study [25].

The active older group demonstrated higher transfer of unesterified cholesterol to HDL than the inactive older group, although the transfer of esterified cholesterol was similar in both groups. In previous studies, it was shown that lower unesterified cholesterol transfer was associated with manifested atherosclerotic diseases, such as in precocious CAD and in ischemic cerebrovascular events (for review, see [3]). Furthermore, in aged individuals and in patients with type 2 diabetes, the unesterified cholesterol transfer to HDL was lower in those with CAD compared with those without CAD [4]. Therefore, the high transfer of unesterified cholesterol to HDL found in the active older group is suggestive that regular physical activity can favor the mechanisms of HDL protection against atherogenesis. However, it is important to mention that, in accordance with the literature, HDL-C is a complex lipoprotein that can be useful as a biomarker in CAD risk, but its direct causal role in CAD remains controversial [26].

The HDL fraction is the main uptake site for cholesterol esterification in the plasma, a fundamental process for cholesterol homeostasis: unesterified cholesterol from the apolipoprotein B-containing lipoproteins and from the peripheral tissues is transferred to HDL. In HDL, cholesterol is esterified by the action of LCAT associated with the HDL fraction, using apolipoprotein A-I as a co-factor. In previous studies, CAD patients showed increased removal of unesterified cholesterol from the plasma, suggesting that this lipid would dissociate from the lipoprotein particle and independently precipitate in the arterial wall [3]. This finding highlights the importance of unesterified cholesterol transfer to HDL fractions as a mechanism that stabilizes the plasma cholesterol pool. Therefore, the finding of a higher transfer of unesterified cholesterol to HDL in the active older group than in the inactive older group and in the active young group than in the inactive young group is suggestive that physical activity has a protective action against atherogenesis. By transferring unesterified cholesterol from the other lipoprotein classes to HDL, wherein unesterified cholesterol is esterified and stored in the HDL core, unesterified cholesterol dissociation from the non-HDL lipoproteins and direct deposit in the arterial wall is avoided.

In previous studies, higher unesterified cholesterol transfer to HDL was observed in physically active subjects, such as active aged women [6]. Unesterified cholesterol transfer was increased in patients with metabolic syndrome after they underwent an exercise training program, although HDL-C did not increase [5]. Here, esterified cholesterol transfer to HDL was higher in the active young group than the inactive young group, but in the active older group and the inactive older group, esterified cholesterol transfer was not different. Whether esterified cholesterol transfer could be consistently considered beneficial is difficult to establish in view of the results of previous studies [3].

In a previous study, it was shown that omnivorous individuals had higher cholesterol transfer rates than vegans, whereas in lacto-ovo-vegetarians. transfer values were intermediate between the two [27]. Thus, it is tempting to investigate in future studies whether nutritional habits could influence cholesterol transfer in subjects that engage in regular physical activity in view of the major influence of both diet and physical exercise on human health.

In our current study, the concentration of LCAT was not affected by physical activity, in agreement with previous reports [28,29]. Regarding CETP concentration, the active older group and the inactive older group, as well as the active young group and the inactive young group, did not differ. This is a debatable issue in the literature, since some studies found lower CETP concentrations were associated with exercise training [30], while others did not [29].

As expected, physical activity did not change LDL-C or apolipoprotein B values. However, this does not imply that being physically active does not impact the metabolism of apolipoprotein B-containing lipoproteins. Athletes showed pronouncedly increased removal of an LDL nanoemulsion model compared to sedentary controls, despite LDL-C being equal in both groups [31]. This finding suggests that athletes had accelerated turnover of LDL and exercise training decreased small, dense LDL subfractions [22,32]. Consequently, the shortened residence time of LDL in the plasma exposes the lipoprotein to lower levels of peroxidation or other modifying processes that lead LDL to pro-atherogenic catabolic pathways [33].

It is widely accepted that cardiorespiratory fitness, which is assessed by VO_2_, is not only a pivotal feature of cardiovascular health but is an independent predictor of all-cause mortality [34]. VO_2_ declines over the years [35], but regular engagement in exercise training can attenuate VO_2_ decrease [36], as was also observed in the present study. In fact, active individuals—older or young—showed higher VO_2_peak values than inactive individuals. Additionally, the maintenance of VO_2_ at satisfactory levels for age, according to the equations used here, has an important role in delaying the development of metabolic disorders, such as dyslipidemia [37,38].

## 5. Conclusions

Our results show that, regardless of whether the individuals are younger or older, regular physical activity increases the transfer of cholesterol from the other lipoprotein classes to HDL. Cholesterol transfer to HDL in the plasma is one of the key features of HDL’s metabolism and anti-atherosclerosis function and was tested here by a novel and straightforward in vitro method. Remarkably, regular physical activity, while increasing cholesterol transfer, did not increase the antioxidant function of HDL. This is an important finding because it suggests that the several functions of HDL can be independent of each other. Consequently, to seize a global understanding of HDL’s impact on reducing atherosclerosis risk, it would be necessary to go far beyond the determination of HDL-cholesterol and to systematically evaluate each one of the putative HDL functions.

## Figures and Tables

**Table 1:** Sex distribution, age, anthropometric, and ventilatory data of the study groups.

Parameters	Active Older (n = 24)	Inactive Older (n = 20)	Active Young (n = 39)	Inactive Young (n = 24)	*p*
Sex (M/F, n)	10/14	12/9	22/17	10/14	N.S.
Age (years)	65 (60:80) ^a,b^	67 (60:79) ^c,d^	29 (20:34)	26 (20:35)	<0.0001
Weight (kg)	64.2 ± 11.3	72.2 ± 10.1	70.3 ± 12.1	67.4 ± 15.1	N.S.
BMI (kg/m^2^)	24.7 ± 2.7 ^e^	28.1 ± 4.8 ^c,d^	24.2 ± 2.4	23.5 ± 3.5	<0.0001
WC (cm)	87 (68:113)	96 (73:110) ^c,f^	82 (62:95)	80 (64:111)	<0.0001
WHR (cm)	0.9 (0.8:1.0) ^a^	0.9 (0.8:1.1) ^c,f^	0.8 (0.7:1.0)	0.9 (0.7:1.0)	<0.0001
VO_2_					
Absolute (L/min)	2.0 (1.3:3.2) ^a^	1.8 (1.2:2.7) ^c^	3.5 (2.1:4.4) ^g^	2.3 (1.4:3.8)	<0.0001
VT (mL/kg/min)	22.6 ± 3.0 ^a,h^	18.4 ± 3.7 ^c,i^	32.1 ± 6.6 ^j^	23.0 ± 4.7	<0.0001
Peak (mL/kg/min)	32.6 ± 5.2 ^a,h,k^	27.3 ± 5.9 ^c,d^	46.4 ± 4.9 ^j^	37.5 ± 7.6	<0.0001
Predicted for age (%)	119 (86:154) ^b,h^	102 (75:130) ^i^	109 (91:129) ^j^	88 (66:170)	<0.0001
Slope (VE/VO_2_)	31 (24:38)	31 (23:41)	29 (23:37)	32 (22:37)	N.S.
MET	9.3 ± 1.5 ^a,h^	7.8 ± 1.7 ^c,d^	13.4 ± 1.6 ^j^	10.5 ± 1.7	<0.0001
OUES (mL)	2339 ± 598 ^a^	2117 ± 405 ^c^	3561 ± 837 ^j^	2602 ± 763	<0.0001

Data are expressed as mean ± standard deviation or median (minimum:maximum). Abbreviations: BMI: body mass index; WC: waist circumference; WHR: waist–hip ratio; VO_2_: oxygen consumption; VT: ventilatory threshold; MET: metabolic equivalent; OUES: oxygen uptake efficiency slope. ^a^—active older vs. active young: *p* < 0.001; ^b^—active older vs. inactive young: *p* < 0.001; ^c^—inactive older vs. active young: *p* < 0.001; ^d^—inactive older vs. inactive young: *p* < 0.001; ^e^—active older vs. inactive older: *p* < 0.01; ^f^—inactive older vs. inactive young: *p* < 0.01; ^g^—active young vs. inactive young: *p* < 0.01; ^h^—active older vs. inactive older: *p* < 0.05; ^i^—inactive older vs. inactive young: *p* < 0.05; ^j^—active young vs. inactive young: *p* < 0.001; ^k^—active older vs. inactive young: *p* < 0.05. N.S.: not significant.

**Table 2:** Plasma lipids, apolipoproteins, and structural and functional HDL parameters of the study groups.

Parameters	Active Older (n = 24)	Inactive Older (n = 21)	Active Young (n = 39)	Inactive Young (n = 24)	*p*
Cholesterol (mg/dL)					
Total	213 ± 32 ^a,b^	203 ± 31 ^c,d^	178 ± 30	163 ± 32	<0.0001
LDL	127 ± 26 ^e,f^	127 ± 24 ^g,h^	103 ± 28	99 ± 26	<0.0001
HDL	67 ± 14 ^b,i^	51 ± 14 ^c^	62 ± 15 ^j^	49 ± 14	<0.0001
non-HDL	146 ± 32 ^e,f^	152 ± 28 ^d,k^	116 ± 29	114 ± 30	<0.0001
Triglycerides (mg/dL)	79 (29:250)	122 (34:217) ^k,h^	64 (24:115)	67 (20:227)	0.0004
Apolipoprotein (g/L)					
A-I	1.69 ± 0.18 ^b^	1.55 ± 0.27 ^j^	1.58 ± 0.27 ^l^	1.34 ± 0.26	<0.0001
B	0.98 ± 0.20 ^b,e^	1.01 ± 0.21 ^d,k^	0.80 ± 0.18	0.76 ± 0.16	<0.0001
HDL size (nm)	9.2 (8.5:9.7)	9.1 (8.7:9.7)	9.2 (8.6:10.0)	9.2 (8.5:9.5)	N.S.
CETP (ug/mL)	1.08 (0.53:2.31) ^a,f^	0.89 (0.42:1.44)	0.58 (0.15:1.53)	0.74 (0.26:1.43)	<0.0001
LCAT (ug/mL)	8.64 ± 1.80	8.01 ± 2.84	7.60 ± 1.19	7.97 ± 1.88	N.S.
Cholesterol transfer to HDL (%)					
Esterified	4.71 (3.62:5.62) ^b^	4.20 (3.18:5.91)	4.23 (3.44:6.06) ^m^	3.83 (2.68:5.03)	0.0002
Unesterified	6.43 (4.83:8.94) ^b,i^	5.45 (3.84:8.20)	5.98 (4.35:9.47) ^m^	5.32 (3.16:7.28)	0.0001

Data are expressed as mean ± standard deviation or median (minimum:maximum). Abbreviations: HDL: high-density lipoprotein; LDL: low-density lipoprotein; CETP: cholesteryl ester transfer protein; LCAT: lecitin–cholesterol acyltransferase; ^a^—active older vs. active young: *p* < 0.001; ^b^—active older vs. inactive young: *p* < 0.001; ^c^—inactive older vs. active young: *p* < 0.05; ^d^—inactive older vs. inactive young: *p* < 0.001; ^e^—active older vs. active young: *p* < 0.01; ^f^—active older vs. inactive young: *p* < 0.01; ^g^—inactive older vs. active young: *p* < 0.01; ^h^—inactive older vs. inactive young: *p* < 0.01; ^i^—active older vs. inactive older: *p* < 0.01; ^j^—active young vs. inactive young: *p* < 0.01; ^k^—inactive older vs. active young: *p* < 0.001; ^l^—inactive older vs. inactive young: *p* < 0.05; ^m^—active young vs. inactive young: *p* < 0.05. N.S.: not significant.

**Table 3:** PON1 activity and HDL anti-oxidant capacity of study groups.

Parameters	Active Older (n = 24)	Inactive Older (n = 21)	Active Young (n = 39)	Inactive Young (n = 24)	*p*
PON1 activity (U/L)	77 (22:143)	46 (11:185)	65 (18:194)	71 (10:173)	N.S.
Lag time (min)	93 (75:115)	92 (78:112)	92 (79:102)	92 (83:116)	N.S.
ODmax.	0.9418 ± 0.0281	0.9330 ± 0.0234	0.9357 ± 0.0275	0.9401 ± 0.0257	N.S.
Tmax (min)	133 (120:155)	133 (110:153)	135 (110:143)	135 (128:163)	N.S.
Vmax (milli-units/min)	11.49 (8.34:12.84)	10.44 (7.89:12.47)	10.94 (8.17:12.84)	10.12 (6.58:12.77)	N.S.
Time to Vmax (min)	90 (83:115)	89 (74:103)	91 (76:103)	90 (80:104)	N.S.

Data are expressed as median (minimum:maximum). Abbrevitation: PON1: paraoxonase 1; ODmax: maximum quantity of conjugated dienes generated; Tmax: time needs for maximum production of conjugated dienes; Vmax: maximum rate of conjugated dienes obtained from the slope of the absorbance curve during the propagation phase. N.S.: not significant.

## Data Availability

The data presented in this study are available on request from the corresponding author.

## Supplementary Materials

The following supporting information can be downloaded at: https://www.mdpi.com/article/10.3390/nu15234871/s1, Table S1. Correlation analysis of data with all participants.
