# Supplementary material for: Regular Practice of Physical Activity Improves Cholesterol Transfers to High-Density Lipoprotein (HDL) and Other HDL Metabolic Parameters in Older Adults
Source: Nutrients. 2023 Nov 22;15(23):4871. doi: 10.3390/nu15234871 (PMC10708266; doi:10.3390/nu15234871)
Supplement: Supplementary file 1 [file nutrients-15-04871-s001.zip › nutrients-2657919-supplementary.pdf]

Supplementary Table S1. Correlation analysis of data with all participants

Parameters		BMI	LCAT	CETP	PON1	HDL size	Small HDL	Inter HDL	Large HDL	EC transfer	UC transfer	VO_2peak
Total cholesterol	r	0.1441	**0.3862**	0.1809	0.1681	0.0845	**0.2341**	0.1621	**0.3723**	**0.4978**	**0.3581**	**-0.3781**
	p	N.S.	**<0.0001**	N.S.	N.S.	N.S.	**0.0148**	N.S.	**<0.0001**	**<0.0001**	**<0.0001**	**<0.0001**
LDL-C	r	**0.2315**	**0.3363**	0.1645	0.0982	0.0329	0.0685	-0.0916	0.1132	**0.3024**	0.1131	**-0.3447**
	p	**0.0159**	**0.0005**	N.S.	N.S.	N.S.	N.S.	N.S.	N.S.	**0.0015**	N.S.	**0.0003**
HDL-C	r	**-0.2770**	0.1342	-0.0026	0.1346	**0.2006**	**0.5702**	**0.7035**	**0.8075**	**0.4597**	**0.5415**	0.0112
	P	**0.0037**	N.S.	N.S.	N.S.	**0.0374**	**<0.0001**	**<0.0001**	**<0.0001**	**<0.0001**	**<0.0001**	N.S.
Non-HDL-C	r	**0.2954**	**0.3645**	0.1884	0.1142	0.0072	0.0108	-0.1505	0.0289	**0.3363**	0.1249	**-0.4109**
	P	**0.0019**	**0.0002**	N.S.	N.S.	N.S.	N.S.	N.S.	N.S.	**0.0004**	N.S.	**<0.0001**
TG	r	**0.2755**	**0.3263**	0.1821	0.0291	-0.1178	**-0.1986**	**-0.2655**	**-0.1977**	**0.2597**	0.1804	**-0.3558**
	p	**0.0039**	**0.0008**	N.S.	N.S.	N.S.	**0.0393**	**0.0055**	**0.0403**	**0.0067**	N.S.	**0.0002**
Apo A-I	r	**-0.1978**	0.1915	0.0012	0.1196	**0.1956**	**0.5093**	**0.5682**	**0.7889**	**0.6168**	**0.6652**	-0.1663
	P	**0.0411**	N.S.	N.S.	N.S.	**0.0435**	**<0.0001**	**<0.0001**	**<0.0001**	**<0.0001**	**<0.0001**	N.S.
Apo B	r	**0.2703**	**0.3822**	**0.2233**	0.0848	-0.0106	-0.0291	-0.1344	-0.0173	**0.3159**	0.1327	**-0.3630**
	p	**0.0049**	**<0.0001**	**0.0208**	N.S.	N.S.	N.S.	N.S.	N.S.	**0.0009**	N.S.	**0.0001**
VO_2peak	r	**-0.2920**	-0.1804	**-0.2417**	-0.0822	0.0439	0.0336	0.0661	-0.0940	-0.1698	-0.0092	-
	p	**0.0023**	N.S.	**0.0121**	N.S.	N.S.	N.S.	N.S.	N.S.	N.S.	N.S.	-
UC transfer	r	-0.1321	**0.4060**	**0.2467**	0.1684	**0.3315**	**0.2365**	**0.4843**	**0.4098**	**0.8685**	-	-
	p	N.S.	**<0.0001**	**0.0100**	N.S.	**0.0005**	**0.0137**	**<0.0001**	**<0.0001**	**<0.0001**	-	-
EC transfer	r	0.0684	**0.3641**	**0.3137**	**0.2020**	**0.3471**	0.1366	**0.3910**	**0.4447**	-	-	-
	p	N.S.	**0.0002**	**0.0009**	**0.0361**	**0.0002**	N.S.	**<0.0001**	**<0.0001**	-	-	-
Large HDL	r	-0.1393	0.0397	-0.0533	0.0964	0.1351	**0.2988**	**0.4368**	-	-	-	-
	p	N.S.	N.S.	N.S.	N.S.	N.S.	**0.0017**	**<0.0001**	-	-	-	-
Intermediate HDL	r	**-0.1976**	**0.2066**	0.1778	0.0678	0.0598	-0.0049	-	-	-	-	-
	p	**0.0404**	**0.0372**	N.S.	N.S.	N.S.	N.S.	-	-	-	-	-
Small HDL	r	**-0.1928**	0.0180	-0.1251	0.1461	**0.1901**	-	-	-	-	-	-
	p	**0.0456**	N.S.	N.S.	N.S.	**0.0488**	-	-	-	-	-	-
HDL size	r	-0.0434	0.1023	-0.1033	0.0661	-	-	-	-	-	-	-
	p	N.S.	N.S.	N.S.	N.S.	-	-	-	-	-	-	-
PON1	r	0.0995	0.0919	-0.1259	-	-	-	-	-	-	-	-
	p	N.S.	N.S.	N.S.	-	-	-	-	-	-	-	-
CETP	r	0.0989	**0.3385**	-	-	-	-	-	-	-	-	-
	p	N.S.	**0.0005**	-	-	-	-	-	-	-	-	-
LCAT	r	-0.0399	-	-	-	-	-	-	-	-	-	-
	p	N.S.	-	-	-	-	-	-	-	-	-	-

Abbreviations: apo: apolipoprotein; BMI: body-mass index; LCAT: lecitin-cholesterol acyltransferase; CETP: cholesteryl ester transfer protein; PON1: paraoxonase 1; HDL: high-density lipoprotein; Inter: intermediate; EC: esterified cholesterol; UC: unesterified cholesterol; VO_2: oxygen consumption.
